# Effect of genotype on individual response to the pharmacological treatment of glaucoma: a systematic review and meta-analysis

**DOI:** 10.1186/s13062-023-00423-4

**Published:** 2023-10-13

**Authors:** Damiana Scuteri, Giulio Pocobelli, Yoichi Sakurada, Rossella Russo, Paolo Tonin, Pierluigi Nicotera, Giacinto Bagetta, Maria Tiziana Corasaniti, Carlo Nucci

**Affiliations:** 1 Pharmacotechnology Documentation and Transfer Unit, Preclinical and Translational Pharmacology, Department of Pharmacy, Health and Nutritional Sciences, University of Calabria, 87036 Rende, Italy; 2 Regional Center for Serious Brain Injuries, S. Anna Institute, 88900 Crotone, Italy; 3 Ophthalmology Unit, Department of Experimental Medicine, University of Rome Tor Vergata, Via Montpellier 1, 00133 Rome, Italy; 4 Department of Ophthalmology, Faculty of Medicine, University of Yamanashi, Chuo, Yamanashi 409-3821 Japan; 5 Preclinical and Translational Pharmacology, Department of Pharmacy, Health and Nutritional Sciences, University of Calabria, 87036 Rende, Italy; 6 German Center for Neurodegenerative Diseases (DZNE), 53127 Bonn, Germany; 7 Department of Health Sciences, University “Magna Graecia” of Catanzaro, 88100 Catanzaro, Italy

**Keywords:** Primary open-angle glaucoma (POAG), Genetic variants, PRISMA 2020, Pharmacological therapy, Efficacy, Safety

## Abstract

The social impact of glaucoma is worth of note: primary open-angle glaucoma (POAG) is one of the leading causes of irreversible blindness worldwide, affecting some 68.56 million people with overall prevalence of 2.4%. Since one of the main risk factors for the development of POAG is the increase of intraocular pressure (IOP) causing retinal ganglion cells death, the medical treatment of POAG consists in the use of drugs endowed with neuroprotective effect and able to reduce IOP. These drugs include beta-blockers, prostaglandin analogues, carbonic anhydrase inhibitors, alpha or cholinergic agonists and rho kinase inhibitors. However, not all the patients respond to the same extent to the therapy in terms of efficacy and safety. Genetics and genome wide association studies have highlighted the occurrence of mutations and polymorphisms influencing the predisposition to develop POAG and its phenotype, as well as affecting the response to pharmacological treatment. The present systematic review and meta-analysis aims at identifying genetic variants and at verifying whether these can influence the responsiveness of patients to therapy for efficacy and safety. It follows the most updated Preferred Reporting Items for Systematic reviews and Meta-Analyses 2020 recommendations. The literature search was conducted consulting the most relevant scientific databases, i.e. PubMed/MEDLINE, Scopus, Web of Science and Public Health Genomics and Precision Health Knowledge Base up to June 14th, 2023. The search retrieved 1026 total records, among which eight met the eligibility criteria for inclusion in the analysis. The results demonstrated that the most investigated pharmacogenetic associations concern latanoprost and timolol, and that efficacy was studied more in depth than safety. Moreover, the heterogeneity of design and paucity of studies prompt further investigation in randomized clinical trials. In fact, adequately powered and designed pharmacogenetic association studies are needed to provide body of evidence with good certainty for a more appropriate use of medical therapy in POAG.

*PROSPERO registration*: CRD42023434867.

## Background

Glaucoma encompasses a group of progressive optical nerve neuropathies characterized by a degeneration of retinal ganglion cells (RGCs) and retinal nerve fiber layers [1], that has a remarkable social impact since it is the leading cause of irreversible blindness worldwide [2]. In particular, primary open-angle glaucoma (POAG) affects some 52.68 million people globally and this number is estimated to increase up to 79.76 million in 2040 [3, 4] due to aging. The social burden of glaucoma is increased by the under and late diagnosis, also due to preperimetric glaucoma devoid of significant functional impairment, leading to irreversible vision loss and reduced quality of life [1]. In fact, it can be asymptomatic until late severe stages [5, 6]. Its pathogenesis is not completely unraveled, but one of the most important risk factors is the increase of intraocular pressure (IOP), in spite of the occurrence of normal tension glaucoma [7]. Glaucoma is anatomically classified in open-angle and angle closure, that, when occurring without an identifiable cause, are primary [8]. POAG is furtherly classified according to the age of onset as primary congenital glaucoma (up to 3 years of age), juvenile open-angle glaucoma (JOAG with onset at 3–35 years), and adult-onset POAG (with onset after 35 years) [9, 10]; the latter is the most common form. The levels of IOP are determined by the balance between secretion of aqueous humor by the ciliary body and its drainage, that can occur through the trabecular meshwork and the uveoscleral outflow pathway: the site of damage to nerve fibers is the scleral lamina cribrosa, fundamental in the degree of susceptibility to damage by elevated IOP [11]. The genetics of glaucoma is very complex. Traditional linkage analysis highlighted through positional cloning that myocilin (MYOC) gene is involved in the development of POAG [12]. Moreover, due to the unraveled physiopathology of glaucoma, genome-wide association studies (GWAS) for POAG were performed, detecting sequence variants and genetic loci encoding for proteins expressed in the trabecular meshwork and RGCs associated with POAG susceptibility in Iceland population [13] and also involved in the pathogenetic mechanisms in Japanese people [14]. Uncommon mutations in the gene encoding neurotrophin-4 (NTF4), causing decreased affinity for its specific tyrosine kinase receptor B (TrkB) that is neuroprotective for RGCs, were highlighted both in European [15] and Chinese [16] populations. Furthermore, a study performed on 54 families with autosomal dominantly inherited adult-onset POAG led to the identification of sequence alterations in the gene OPTN of optineurin, expressed in trabecular meshwork, nonpigmented ciliary epithelium, retina, and brain [17]. The WD40-repeat 36 gene was found in patients suffering from high and low-pressure POAG [18]. The purpose of the pharmacological treatment of POAG consists in the reduction of IOP and overall neuroprotection to prevent RGC death [19, 20], thus proposing antioxidants as well [21]. In many patients lowering the IOP by ≥ 25% slows down the progression of glaucoma, as demonstrated in the Early Manifest Glaucoma Trial [22]. The classes of topical pharmacological therapies for glaucoma include: prostaglandin analogues (e.g. latanoprost, bimatoprost and travoprost), beta-blockers (e.g. timolol), alpha-adrenergic agonists (as brimonidine [23]), carbonic anhydrase inhibitors (e.g. brinzolamide and dorzolamide), cholinergic agonists (as pilocarpine) and Rho kinase inhibitors (ripasudil and netarsudil, that are thought to decrease episcleral venous pressure, fibrosis and the production of aqueous humor reducing IOP [24]). Apart from the susceptibility to develop glaucoma and towards a more severe progression of the disease, the inter-individual variation in drug response and in the occurrence of adverse drug reactions has been gaining interest over the last years, as for other neurological diseases characterized by resistance to treatment [25, 26]. Pharmacogenetic assessments demonstrated an increased risk of developing steroid-induced ocular hypertension after treatment with prednisolone acetate following photorefractive keratectomy associated to the variant N363S of glucocorticoid receptor [27]. Also, the CC genotype of the single nucleotide polymorphism (SNP) rs1042714 of the adrenergic beta2 receptor gene ADRB2 responds to topical beta-blockers, as timolol, with more significant reduction of IOP [28], while the CC genotype of the polymorphism R296C of the cytochrome CYP2D6 does not develop timolol-induced bradycardia [29] and CYP2D6 poor metabolizers may present more frequently systemic adverse events [30]. Pharmacogenetic evaluations were conducted for the response to latanoprost pointing at the correlation of low responders to IOP decrease with the SNP rs 3753380 of the prostaglandin F (2 alpha) receptor in patients with glaucoma and ocular hypertension [31]. Therefore, the aim of the present study is to provide for the first time a comprehensive systematic review and meta-analysis of role of genetic variants in the response to all the phamacological treatments available for POAG in terms of efficacy and safety. This systematic review and meta-analysis is registered in the National Institute for Health Research (NIHR) International prospective register of systematic reviews (PROSPERO) with number CRD42023434867.

## Methods

### Objectives, registration and protocol

Systematic literature search, screening of retrieved records and selection of the results meeting the inclusion criteria followed the most recently updated Preferred Reporting Items for Systematic reviews and Meta-Analyses (PRISMA) 2020 recommendations [32–34] and the guidance from the Human Genome Epidemiology Network for reporting gene-disease associations [35] to answer to the PICOS (participants/population, interventions, comparisons, outcomes, and study design) question formulated to understand whether the different genotypes and microRNAs (miRNAs) affect the efficacy and safety of pharmacological therapies to treat patients of any age and ethnicity affected by POAG. Study designs deemed to be eligible include both clinical trials and any type of observational study as studies investigating direct genetic association. In vivo and in vitro preclinical studies, reviews, book chapters and congress communications and proceedings are excluded. Studies not available in full text in English were excluded. The protocol was set a priori to the literature search and registered in PROSPERO (CRD42023434867).

### Information sources

The literature search was performed inspecting the most relevant scientific databases, i.e. PubMed/MEDLINE, Scopus, Web of Science (WOS) and Public Health Genomics and Precision Health Knowledge Base (PHGKB) from database inception up to the date of last search that is June 14th, 2023. No restriction of publication date has been applied.

### Search strategy

The following medical and subject headings (MeSH) terms, keywords and modifications were combined in search strings using the Boolean operator “AND”: “primary open-angle glaucoma”, “genetics”, “genotypes”, “polymorphisms”, “SNPs”, “miRNAs”, “mutations”, “pharmacological therapy”, “prostaglandin analog(ue)s”, “beta(-)blockers”, “alpha agonists”, “carbonic anhydrase inhibitors”, “cholinergic agonists”, “rho kinase inhibitors”, “Glaucoma, Open-Angle/genetics”[Mesh], “Glaucoma, Open-Angle/therapy”[Mesh], “glaucoma”, “therapy”, “genetics”. A high sensitivity/recall search strategy that can maintain precision was used [36]*.*

### Selection of the studies and extraction of data

Studies were selected based on the assessment of eligibility criteria, conducted by two independent authors to minimize the risk of excluding relevant records. Lines and spelling of strings and the suitability of the search to cover all the most relevant literature to answer to the PICOS question were revised by an author different (reviewer) from the two consulting independently the databases (requestors), in accordance with the evidence-based guideline for Peer Review of Electronic Search Strategies (PRESS) for systematic reviews (SRs) [36, 37]. Duplicate records were removed by reference manager software (EndNote X7, Clarivate). The following first screening consisted in the evaluation of title and abstract. Then, the full text was assessed for inclusion. The references list of the articles was inspected to extend and refine the search. Complete consensus among all the authors was achieved without relevant conflicts, planned to be solved through consensus or consulting a third committee member. Data were extracted from text, tables or graphs of the included records.

### Data synthesis, assessment of the risk of bias and critical appraisal

The synthesis of the results followed the Cochrane Consumers and Communication Review Group guidelines [38]. The assessment of the risk of bias (RoB) and of the quality of retrieved studies was conducted according to Human Genome Epidemiology (HuGE) systematic reviews and meta-analyses risk-of-bias score for genetic association studies [39] taking into account the following domains: (1) Information bias—Accuracy of diagnosis and robustness of genotyping methods; (2) Confounding bias—Population stratification and other confounder effects; (3) Selective reporting of outcomes—reporting bias; (4) Hardy–Weinberg equilibrium (HWE)—assessment in the control groups. The graphical representation of the RoB assessment was produced using the Cochrane robvis visualization tool [40].

### Statistical analysis and effect measures

The Cochrane Review Manager 5.4.1 (RevMan5.4.1; Copenhagen: The Nordic Cochrane Center, The Cochrane Collaboration) was used to measure relative risks (RR) and 95% confidence intervals (CI) or standardized mean differences (SMD) and inverse variance for dichotomous and continuous variables, respectively. The heterogeneity was calculated through the random effect model [41] and the Higgins I^2^ value [42]. Egger’s linear regression test was used to assess publication bias [43].

## Results

### Studies selection

**Fig. 1:**
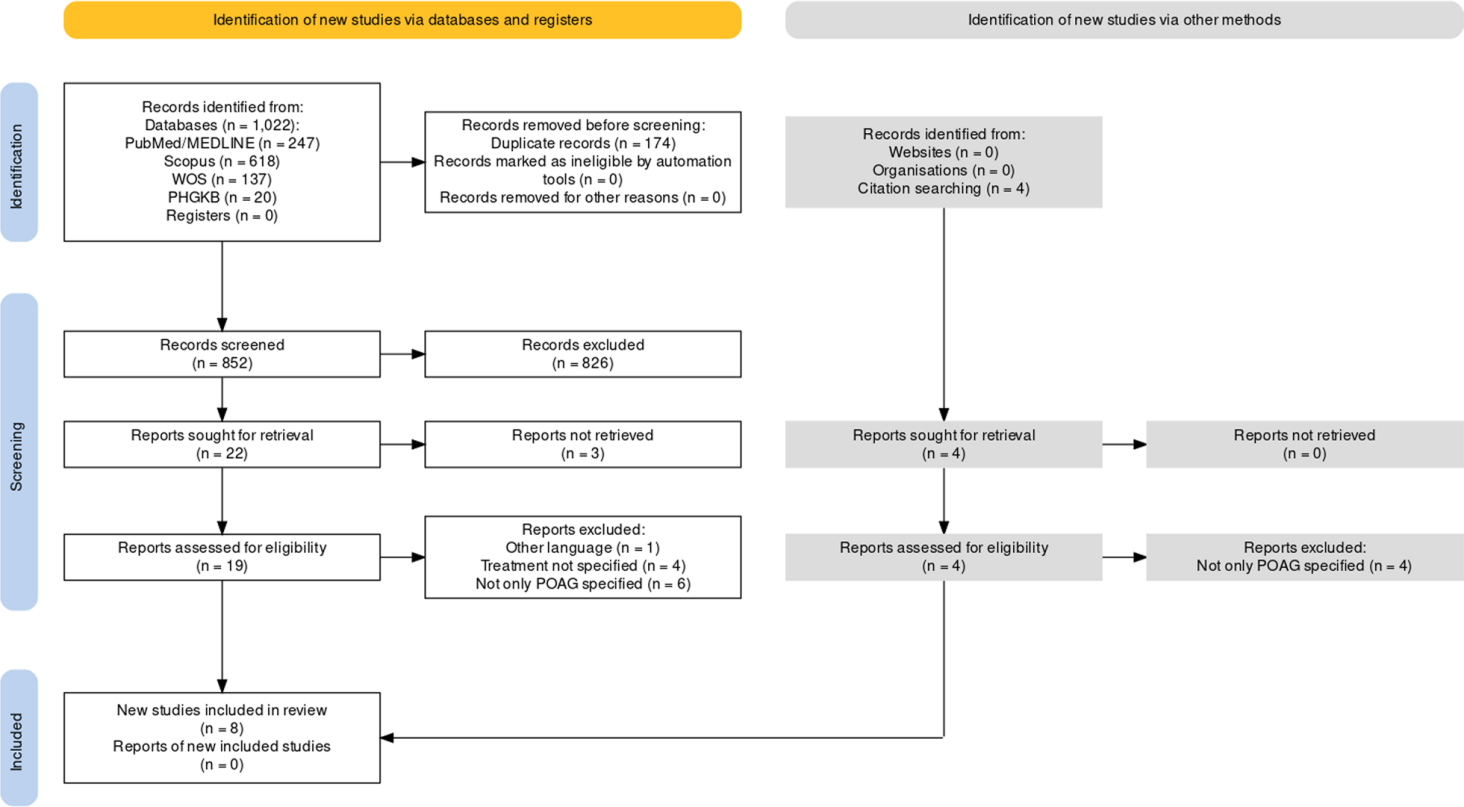
PRISMA flow diagram. Selection of records based on the Preferred Reporting Items for Systematic Reviews and Meta-Analyses (PRISMA) 2020. Flow diagram produced with the web-based Shiny app [66]

The search of PubMed/MEDLINE retrieved 247 records. Other 618 records were obtained from Scopus screening, 137 from WOS and 20 from PHGKB. Four results were found from inspection of the references list of articles. Therefore, the search retrieved a total of 1026 records. The removal of duplicates left 852 records to screen. The screening of title and abstract caused the exclusion of all the studies that did not meet the inclusion criteria for different outcomes investigated or study design, etc. Twenty-six records remained to be examined and were sought for retrieval. The full text was not available for the following 3 articles: Campos-Mollo et al. [44], Lei et al. [45], Moshetova et al. [46]. The report by Kirilenko et al. [47] was excluded because the article was written in Russian. The study by Pleet et al. [48] was not eligible since the treatment was not specified, as it occurs in the studies by Qassim et al. [49], by Wei et al. [50] and by Zebardast et al. [51]. The studies by McCarty et al. [28], by Salminen et al. [52], by Sakurai et al. [31] and by Nieminen et al. [30] had to be excluded because POAG was not reported as disease affecting the population object of study. The paper by Hedman et al. [53] was excluded since it included also ocular hypertension apart from POAG and the study by Netland et al. [54] was excluded because the population included also sufferers from pseudoexfoliative glaucoma. The study by Canut et al. [55] aimed at predicting the individual response to ocular hypotensive drugs, but including both POAG and ocular hypertension, thus it had to be excluded from the analysis. Also, the study by Zhang et al. [56] and by McCarty et al. [57] included patients with ocular hypertension, thus being excluded. Due to the use of multiple medications, representing a different study design, the study by Opazo-Toro et al. [58] could not be included in the meta-analysis. In particular, the paper by Opazo-Toro et al. [58] included also ocular hypertension and showed more severe glaucoma and impairment of visual field in agreement with significantly higher IOP after treatment with beta-blockers and/or prostaglandin analogues and other types of ocular hypotensive treatments (*P* = 0.031). Full text screening left 8 results eligible for inclusion in the analysis. The process of database search and selection of studies is illustrated in Fig. 1 and the most relevant features of the studies included are reported in Table 1.

**Table 1:** Main characteristics of the studies included in the analysis

Study	Design	Population	Control	Ethnicity	Hardy–Weinberg equilibrium (HWE)	Variant	Intervention	Results
Colomb et al. [59]	Retrospective	Patients affected by primary open-angle glaucoma (POAG) diagnosed by the conjunction of a characteristic cupping of the optic disk, an open iridocorneal angle (grade III or IV gonioscopy), and an alteration of the visual field, tested by automated perimetry (with Humphrey’s perimeter or Octopus). Elevated intraocular pressure (IOP) > 21 mmHg by applanation tonometry on at least two examinations. N = 117	All baseline differences were not statistically signifcant, apart from IOP and visual field. Total controls N = 94; allele control N = 25	French	Not reported	Trabecular meshwork-inducible glucocorticoid response (TIGR)/MYOCILIN (MYOC) MYOC.mt1	Topical beta-blockers that could be associated with miotics	Slower decrease of IOP in comparison with the non carriers of the allele and female patients did not show any reduction of IOP
Cui et al. [60]	Prospective study	POAG was defined by the criteria of the International Society of Geographic and Epidemiological Ophthalmology (ISGEO) [7]: an untreated Intraocular pressure (IOP) of 21 mmHg or more with a Goldman applanation tonometery, open anterior chamber angles on gonioscopy; glaucomatous optic disc changes (increased cup/disc ratio, thinning of the neuroretinal rim, notching) on ophthalmoscopy and visual field defects characteristic of glaucoma by standard automated perimetry with the Humphrey Visual Field Analyzer. N = 135 divided per each genotype	PTGFR rs3766355 A > C was associated to higher pre-treatment IOP (*P* < 0.05)	Not reported	HWE was tested by chi-square test and genotypes conformed to it	rs11723068 G > A and rs757253 T > C of the Actin filament-associated protein (AFAP) gene; rs9503012 C > T and rs17134549 T > A of the GDP-mannose 4,6 dehydratase (GMDS) gene; rs3753380 C > T and rs3766355 A > C of the prostaglandin F2 receptor negative regulator (PTGFR)	Latanoprost	TT genotype of GMDS rs9503012 C > T as well as AA genotype of PTGFR rs3766355 A > C was correlated with a statistically significant better response to the therapy with latanoprost. On the contrary, age, CC + CT genotypes of GMDS rs9503012 C > T and CC + AC genotypes of PTGFR rs3766355 A > C were associated with worse response to latanoprost
Gao et al. [61]	Prospective registered in the Chinese Clinical Trial Registry (registration number: ChiCTR-OCC- 11,001,326)	POAG, N = 60 divided per each genotype	No significant baseline differences, as reported in supplementary materials S1 (*P* > 0.05)	Chinese	HWE was analyzed by using Pearson χ2 test of goodness-of-fit in the study sample proving respected	Prostaglandin-endoperoxide synthase 1 (PTGS1) (rs3842787 and rs10306114); PTGFR (rs3753380 and rs3766355); multidrug resistance protein 4 (MRP4) (rs11568658 and rs11568668)	Latanoprost	No difference in frequency and type of side effects after treatment with latanoprost, but 3435C > T (CC and TT mainly) genotype frequency distribution was significantly higher in the group experiencing effectiveness (*P* = 0.002 and *P* = 0.001, respectively)
Liu et al. [62]	Case–control study	The diagnosis of POAG was based on diagnostic criteria published by the Chinese Medical Association Glaucoma Branch in 2008. The criteria for diagnosis of POAG were as follows: (1) IOP ≥ 21 mmHg; (2) abnormal optic disc determined by optical coherence tomography; (3) glaucomatous visual field deletion (on the basis of mean deviation and corrected pattern standard deviation); (4) retinal nerve fiber layer defect; and (5) open anterior chamber angle N = 93	No baseline differences (*P* > 0.05). N = 125	Han subjects in this study were not blood relatives	All patients with different allelic and genotypic frequencies were in HWE	ATP-binding cassette sub-family B member 1 (ABCB1), also known as MRP4	Latanoprost	No difference in the frequency and type of side effects after treatment with latanoprost, but 3435C > T (CC and TT mainly) genotype frequency distribution significantly higher in the group showing efficacy of latanoprost (*P* = 0.002 and *P* = 0.001, respectively)
Liu et al. [63]	Prospective study	POAG defined as early stage, but without defining the criteria. N = 129	One hundred and twenty one age and gender matched healthy people in the same geographical area were randomly selected and identified as the control group. Baseline characteristics do not significantly differ (*P* > 0.05)	Not reported, but Yunnan Province was specified as geographical area	Genotype frequency distributions of ABCB1 gene polymor-phisms-129T > C, 1236C > T, 2677G > T/A and 3435C > T in the case group and the control group were in HWE	Cytochrome P450 2C19 (CYP2C19)	Timolol	In the two groups developing side effects or showing absence of side effects the frequencies of extensive metabolizer phenotype and poor metabolizer phenotype or poor metabolizer phenotype and intermediate metabolizer phenotype were significantly different (both *P* < 0.05), but not between intermediate metabolizer phenotype and extensive metabolizer phenotype (*P* > 0.05). Side effects are more frequent in the group of the poor metabolizers
Ussa et al. [64]	Multicenter case–control study of 5 participating centers	Diagnosis of POAG according to the American Academy of Ophthalmology preferred practice pattern guidelines, optic disc or retinal nerve fiber layer abnormalities, reproducible visual field abnormality and open anterior chamber angles. N = 124	A total of 117 DNA samples could be used for the study: 98 (83.8%) represented the group of responders, among whom 8 (7.7%) were hyperresponders, and 19 (16.2%) were nonresponders. No significant baseline differences apart from IOP	Caucasian, Spanish	HWE was assessed with the Pearson goodness-of-fit test or Fisher exact test when there was a low genotype count. HWE was verified for all the polymorphisms apart from rs7545762 (PTGFR) showing an inconsistent distribution in the nonresponder group	rs6686438 and rs1328441 (PTGFR), rs10489950 and rs3753380 (MMP-1), polymorphisms of MMP-2, -3, -9, and -17	Latanoprost	Polymorphisms of PTGFR, as well as of the gene coding for matrix metalloproteinases 1 (MMP-1), were found to influence the effectiveness of the treatment
Yang et al. [29]	Prospective study	POAG diagnosed as intraocular hypertension (IOP ≥ 21 mmHg), glaucomatous visual fi eld deletion (on the basis of mean deviation and corrected pattern standard deviation, or corrected loss variance of standard 30/II Humphrey perimetry), and abnormal optic disc as determined by the optical coherence tomography. N = 133, but N = 73 were included in genotyping	There were no significant baseline differences among subjects with Arg296Cys or Ser486Thr genotypes (*P* > 0.05)	The genotype frequencies approached corresponding data of Asian people declared on the NCBI Web page (http://www.ncbi.nlm.nih.gov/) in HWE	Genotypes for Pro34Ser were not in HWE	Eight polymorphisms of CYP2D6	Timolol	Genotypes Arg296Cys and Ser486Thr did not significantly affect IOP. Arg296Cys CT and TT genotype were significantly more predisposed to develop bradycardia than CC (*P* = 0.009)
Yuan et al. [65]	Prospective study	POAG N = 123		Not reported		rs16947 (2850C > T, R296C) and rs1135840 (4180C > G, S486T) polymorphims of CYP2D6	Timolol	rs16947 (2850C > T, R296C) and rs1135840 (4180C > G, S486T) did not influence the IOP lowering effect induced by timolol (*P* = 0.339 and *P* = 0.903, respectively)

### Data synthesis

#### Beta-blockers

The paper by Colomb et al. [59] reports about a retrospective study investigating the effect of the (− 1000C/G) located in the upstream region of the trabecular meshwork-inducible glucocorticoid response (TIGR)/MYOCILIN (MYOC) gene on POAG phenotype on 142 patients. According to the results, an association was identified mainly in female patients between the G allele (MYOC.mt1) and an increase of IOP (+ 4.9 mmHg, *P* = 0.0004) with a more pronounced impairment of visual field (*P* = 0.02). With regard to the pharmacological response to therapy, male patients presented a slower decrease of IOP in comparison with the non carriers of the allele and female patients did not show any reduction of IOP. The pharmacological therapy included primarily topical beta-blockers that could be associated with miotics. The study by Liu et al. [62] assessed the influence of cytochrome P450 2C19 (CYP2C19) polymorphisms on the response to treatment with timolol in terms of both efficacy and safety. Extensive, intermediate and poor metabolizers are not significantly associated to the susceptibility to POAG. In the two groups presenting side effects or showing absence of side effects the frequencies of extensive metabolizer phenotype and poor metabolizer phenotype or poor metabolizer phenotype and intermediate metabolizer phenotype were significantly different (both *P* < 0.05), but not between intermediate metabolizer phenotype and extensive metabolizer phenotype (*P* > 0.05). In particular, side effects are more frequent in the poor metabolizer phenotype group, likely because of delayed metabolism. This is supported by the findings that show worse response to timolol in extensive metabolizers. In the study by Yang et al. [29] 8 SNPs of CYP2D6 were inspected to understand on timolol-induced lowering of IOP and side effects, i.e. bradycardia, demonstrating that the genotypes Arg296Cys and Ser486Thr did not significantly affect IOP. However, Arg296Cys CT and TT genotype were significantly more predisposed to develop bradycardia than the CC genotype (*P* = 0.009). Also, the study by Yuan et al. [65] reported that the CYP2D6 SNPs rs16947 (2850C > T, R296C) and rs1135840 (4180C > G, S486T) did not influence the IOP lowering effect induced by timolol (*P* = 0.339 and *P* = 0.903, respectively), while rs16947 CT (*P* = 0.043) and TT (*P* = 0.043) displayed a predisposition to bradycardia than rs16947 CC, although without significant difference between CT and TT (*P* = 0.177).

#### Prostaglandin analogues

The study of Cui et al. [60] assessed the association of the following SNPs with the pharmacological response to POAG: rs11723068 G > A and rs757253 T > C of the Actin filament-associated protein (AFAP) gene; rs9503012 C > T and rs17134549 T > A of the GDP-mannose 4,6 dehydratase (GMDS) gene; rs3753380 C > T and rs3766355 A > C of the prostaglandin F2 receptor negative regulator (PTGFR). The genotype PTGFR rs3766355 A > C was associated to higher pre-treatment IOP and TT genotype of GMDS rs9503012 C > T as well as AA genotype of PTGFR rs3766355 A > C was correlated with a statistically significant better response to the therapy with latanoprost. On the contrary, age, CC + CT genotypes of GMDS rs9503012 C > T and CC + AC genotypes of PTGFR rs3766355 A > C are linked with worse response to latanoprost. Also the research by Gao et al. [61] investigated the effect on the response to latanoprost of the following polymorphisms: prostaglandin-endoperoxide synthase 1 (PTGS1) (rs3842787 and rs10306114); PTGFR (rs3753380 and rs3766355); multidrug resistance protein 4 (MRP4) (rs11568658 and rs11568668). The results in terms of percent IOP reduction (%ΔIOP) in the treated eye demonstrated significantly lower values in carriers of rs11568658 GT heterozygous genotype, of rs10306114 AG heterozygous genotype and of AT haplotype constructed by rs3753380 and rs3766355. The study of Liu et al. [63] demonstrated that polymorphisms of ATP-binding cassette sub-family B member 1 (ABCB1), also known as MRP4 that was investigated by Gao et al. [61], there was statistically significant difference in frequency between 2677G > T/A and 3435C > T (both P < 0.01), but not for − 129T > C and 1236C > T polymorphisms. Moreover, the frequency of TT + AA + TA mutant genotype of 2677G > T/A and of TT genotype of 3435C > T was significantly higher in the POAG than in the control group (both *P* < 0.01). On the contrary, no difference was reported in the frequency and type of side effects after treatment with latanoprost, but 3435C > T (CC and TT mainly) genotype frequency distribution was significantly higher in the group showing efficacy of latanoprost (*P* = 0.002 and *P* = 0.001, respectively). Also, visual field improvement was significantly correlated with 3435C > T genotype (CT + CC: P < 0.01). Polymorphisms of PTGFR, as well as of the gene coding for matrix metalloproteinases 1 (MMP-1), were found to influence the effectiveness of the treatment with latanoprost in the study by Ussa et al. [64]. The PTGFR polymorphisms showed the following results: rs6686438 and rs1328441 followed an additive inheritance model in which the minor allele increases the possibility of a positive response to latanoprost (odds ratio (OR), 0.2163; 95% confidence interval (CI) 0.0487–0.6363; and OR, 0.3199; 95% CI 0.14–0.6779; respectively); rs10782665 followed a dominant inheritance model for frequent variant increases 3 times the possibility of a positive response (OR, 0.3032; 95% CI 0.1085–0.7161); rs6672484, followed a dominant inheritance model, C/T increases the risk of a nonresponse to latanoprost (OR, 2.4479; 95% CI 1.1891–5.0247); and rs11578155 followed an over dominant model, in which the possibility to be nonresponder to latanoprost is increased 3 times (OR, 2.9119; 95% CI 1.0173–7.6915). In particular, rs10489950 and rs3753380 are near to statically significance (*P* = 0.0534 and *P* = 0.1505, respectively). On the contrary, the MMP-1 gene resulted to have 6 subhaplotypes associated with no response to latanoprost (*P* = 0.01), while MMP-2, -3, -9, and -17 did not affect the response.

### Critical appraisal

**Fig. 2:**
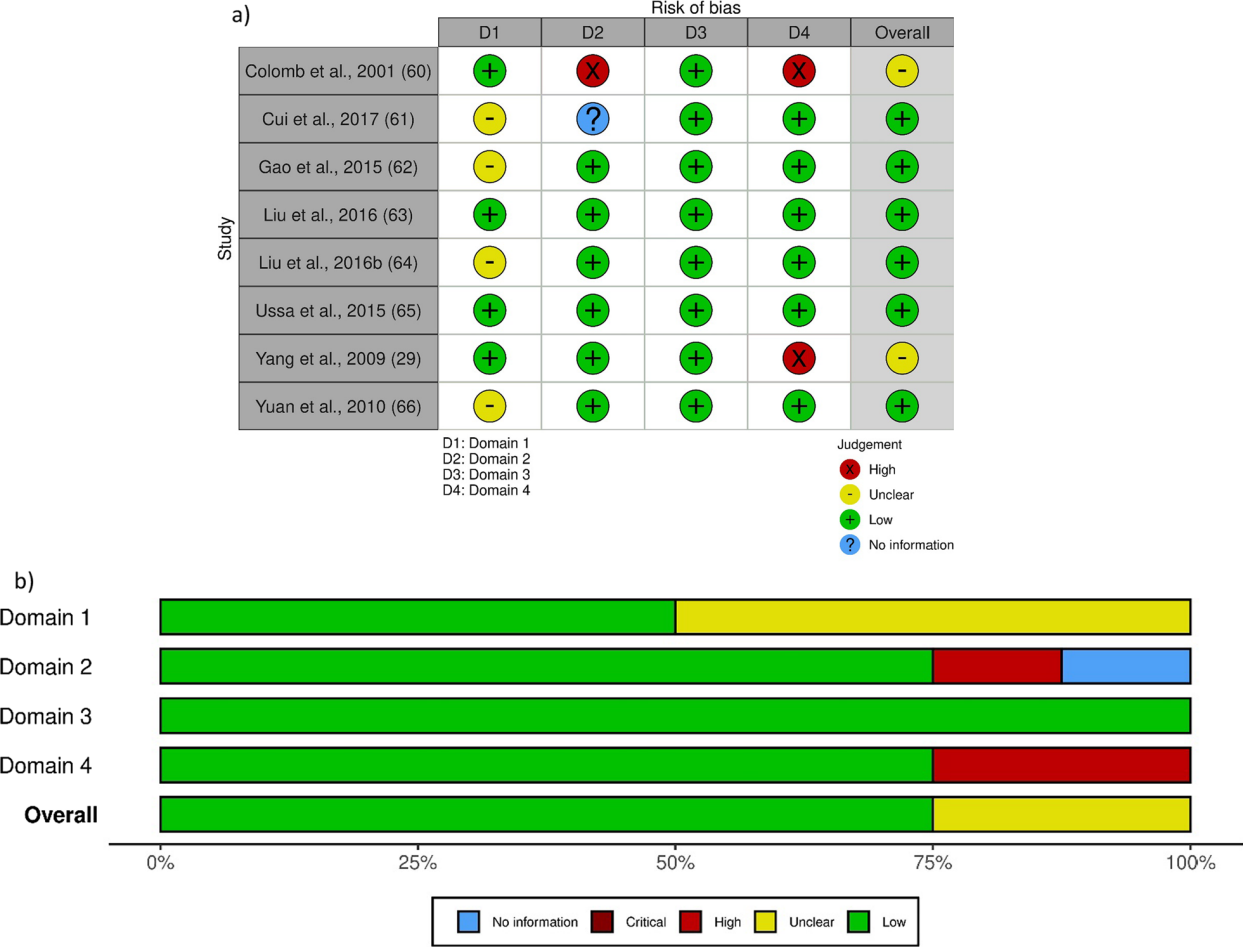
Risk of Bias (RoB) assessment as traffic-light plot (**a**) and weighted bar plots (**b**). The Cochrane robvis visualization tool was used to present RoB [70]

The certainty of evidence based on the studies included in the present systematic review and meta-analysis was assessed following the HuGE systematic reviews and meta-analyses RoB score for genetic association studies [39, 67–69] rating the following 4 outcomes: (1) Information bias, assessing the accuracy of diagnosis of POAG, the ascertainment of controls matched to cases (baseline differences) and the quality of genotyping; (2) Confounding bias, evaluating the possible confounders (population stratification, different ethnicity/gender, sample power calculation and statistical adjustment for confounders); (3) Selective reporting of outcomes, that occurs if only significant associations with SNPs were reported; (4) HWE assessment in the control group of each study. Each of these 4 domains was rated for the presence of low RoB as low risk, high risk, and unclear if insufficient information was available for assessment. Bias assessment is reported in Fig. 2. The study by Colomb et al. [59] presents low RoB for domain 1 since POAG was diagnosed by the conjunction of a characteristic cupping of the optic disk, an open iridocorneal angle (grade III or IV gonioscopy), and an alteration of the visual field, tested by automated perimetry (with Humphrey’s perimeter or Octopus), also presenting elevated IOP > 21 mmHg by applanation tonometry on at least two examinations. In particular, it was clearly defined that patients with a cause of secondary glaucoma were excluded. Baseline differences were not statistically significant, apart from IOP (*P* = 0.0004) and visual field (*P* = 0.02), representing parameters object of the study. The quality of genotyping is guaranteed in the methodology and masking of the operator. RoB arises for domain 2 due to the retrospective nature of the study and to the assessment of visual fields in a non standardized manner, causing that a semi-quantitative grading procedure was used. No selective reporting occurred, but HWE assessment was absent. The study by Cui et al. [60] shows low RoB for domain 1 since POAG was diagnosed by internationally accepted criteria and baseline differences occur only for IOP as in the study by Colomb et al. [59]. The quality of genotyping is guaranteed by the methodology, but ethnicity was not reported. No selective reporting occurred and HWE was conducted with data resulting conform. The study by Gao et al. [61] is a prospective study devoid of reporting bias, in which HWE was analyzed using Pearson χ^2^ test of goodness-of-fit in the study sample resulting respected. Sample power calculation is reported as well as a correct genotyping and the absence of significant baseline differences, as reported in supplementary materials S1 (*P* > 0.05). However, the criteria for POAG diagnosis are not reported. In the study by Liu et al. [62], that is a case–control study, all patients with different allelic and genotypic frequencies were in HWE. The diagnosis of POAG was based on diagnostic criteria published by the Chinese Medical Association Glaucoma Branch in 2008. The criteria for diagnosis of POAG were as follows: (1) IOP ≥ 21 mmHg; (2) abnormal optic disc determined by optical coherence tomography; (3) glaucomatous visual field deletion (on the basis of mean deviation and corrected pattern standard deviation); (4) retinal nerve fiber layer defect; and (5) open anterior chamber angle. No reporting bias occurred and significant baseline differences were not found (*P* > 0.05). In the study by Liu et al. [63] POAG was defined as early stage, but without defining the criteria. A real control group of matched healthy people in the same geographical area were randomly selected. Baseline characteristics did not significantly differ (*P* > 0.05). Case group and the control group were in HWE. In the multicentric study by Ussa et al. [64] patients with very well defined criteria were included among which: Caucasian Spanish origin, diagnosis of POAG according to the American Academy of Ophthalmology preferred practice pattern guidelines, optic disc or retinal nerve fiber layer abnormalities, reproducible visual field abnormality and open anterior chamber angles. HWE was respected for all but one SNP and there were no significant baseline differences apart from IOP. Also, sample power was calculated. In the study by Yang et al. [29] genotypes for Pro34Ser were not in HWE. There were no significant baseline differences among subjects with Arg296Cys or Ser486Thr genotypes (*P* > 0.05). In the study by HWE test demonstrated that all subjects were in equilibrium and there were no statistically significant baseline differences (*P* > 0.05), but the criteria for the diagnosis of POAG were not reported. In the study by Yuan et al., even though the results are reported, thus preventing reporting bias, it is stated that for rs16947 the value of P was obtained by deleting the TT group. Overall, the studies present similar design and certainty of evidence. The RoB graph is illustrated in Fig. 2.

### Meta-analysis

**Fig. 3:**
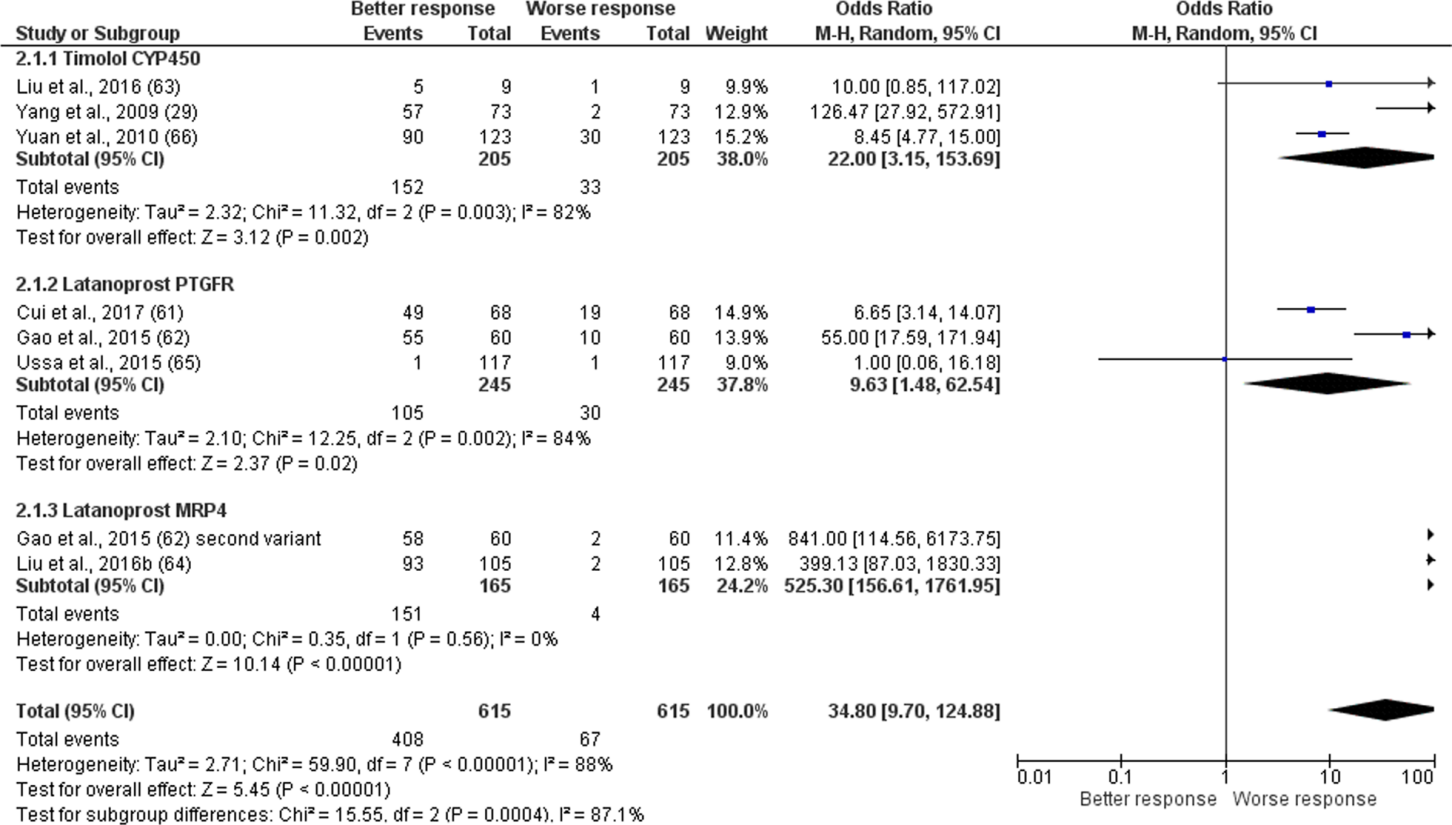
Forest plot for the meta-analysis of the outcome efficacy demonstrating statistically significant effect of polymorphisms of PTGFR (*P* = 0.02) and of MRP4 (*P* < 0.00001) on the efficacy of latanoprost and of polymorphisms of CYP450 on the efficacy of timolol (*P* = 0.002). The total result was statistically significant for the efficacy outcome (OR 34.80 [9.70–124.88], *P* < 0.00001)

**Fig. 4:**
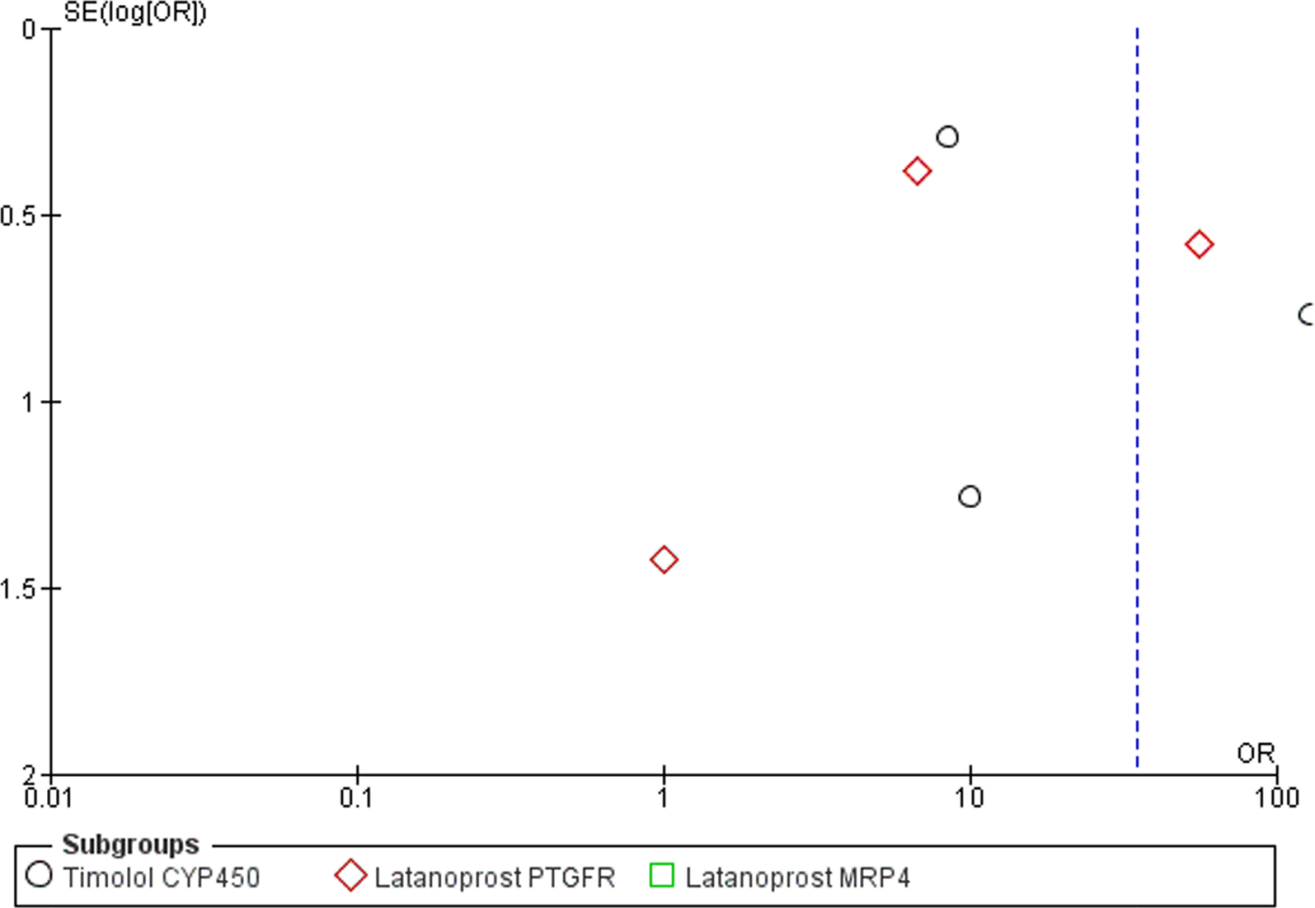
Funnel plot related to the meta-analysis for efficacy outcome. The asymmetry suggests publication bias for the lack of small studies, as supported by the gap in the right bottom figure

The first meta-analysis (forest plot reported in Fig. 3 with subgroup analysis for treatment and genotype) includes all the studies involving the same treatment, i.e. latanoprost and timolol, divided per gene for which genetic variants were examined to assess the influence of genotype on responders and nonresponders to latanoprost. The studies analyzed in the subgroup of latanoprost include all the records investigating the gene PTGFR (Cui et al. [60]; Gao et al. [61]; Ussa et al. [64]) and MRP4 (Gao et al. [61], Liu et al. [62]). The records subjected to subgroup analysis for timolol include the studies assessing genetic variants of CYP450 (Liu et al. [63]; Yang et al. [29]; Yuan et al. [65]). The study by Colomb et al. [59] was excluded from the subgroup of timolol since beta-blockers were used, but the gene investigated encoded for myocilin. A second meta-analysis for the assessment of the effect of the CYP450 variants on safety of timolol was performed. Meta-analysis was performed on n = 615 total patients presenting genetic variants among whom n = 445 treated with latanoprost and n = 165 subjected to treatment with timolol. The meta-analysis for efficacy demonstrates statistically significant effect of polymorphisms of PTGFR (*P* = 0.02) and of MRP4 (*P* < 0.00001) on the efficacy of latanoprost and of polymorphisms of CYP450 on the efficacy of timolol (*P* = 0.002). Only the study by Ussa et al. [64] crossed the line of null effect, influencing the overall result. In agreement with the diamond placement, the total result was statistically significant for the efficacy outcome (*P* < 0.00001), in agreement with the heterogeneity of the studies (I^2^ = 88%; *P* < 0.00001). The funnel plot asymmetry suggests publication bias (Fig. 4) and a gap in the right bottom side of the graph points at smaller studies missing [71].

**Fig. 5:**
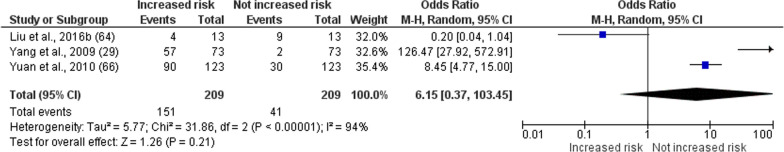
Forest plot for the meta-analysis of the outcome safety demonstrating non statistically significant effect of polymorphisms of cytochrome P450 on the risk to develop bradycardia after treatment with timolol (OR 6.15 [0.37–103.45], *P* = 0.21). Only three studies with high heterogeneity (I^2^ = 94%; *P* < 0.00001) investigated this outcome

**Fig. 6:**
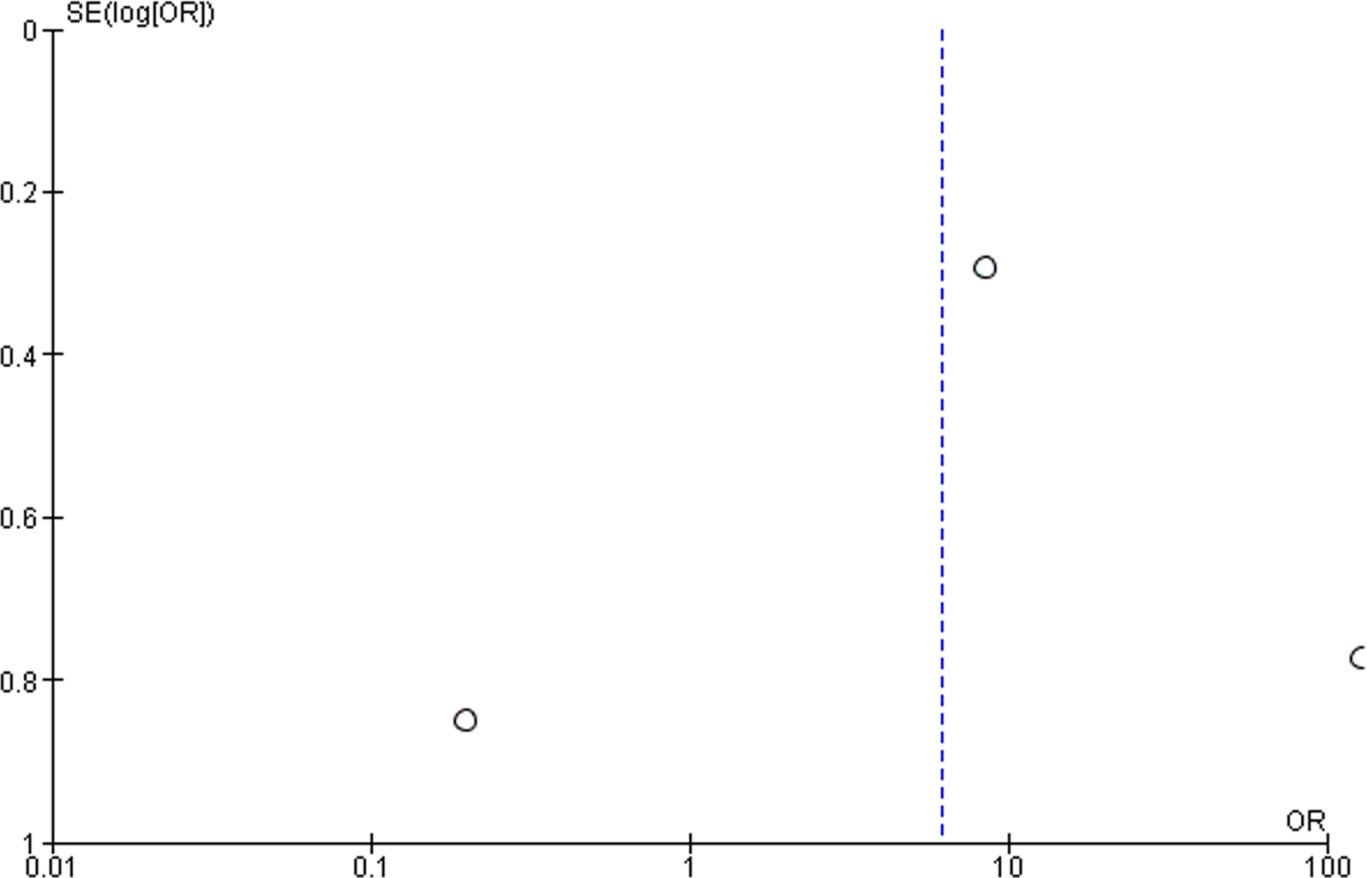
Funnel plot related to the meta-analysis for safety outcome. No significant publication bias is highlighted

The meta-analysis for safety (Fig. 5) shows that the effect of the SNPs of CYP450 on the safety of timolol and, in particular, on the risk to develop bradycardia is not statistically significant (*P* = 0.21). This can be explained by the lack of studies, since the meta-analysis for safety outcome was performed on n = 209 patients subjected to SNPs and treated with timolol. In fact, only three studies with high heterogeneity (I^2^ = 94%; *P* < 0.00001) investigated this outcome. Publication bias is less marked according to the funnel plot (Fig. 6).

## Discussion

POAG is a progressive optic neuropathy often responsible for bilateral irreversible blindness and undiagnosed people can almost equal diagnosed patients suffering from glaucoma [3], thus accounting for the social burden of the disease. The correlation between different genotypes and the particular phenotype of glaucoma was examined in several studies, also to provide reliable genetic models of the disease. It was demonstrated that people of African ancestry are more predisposed to the risk of POAG than people of European ancestry (OR, 2.80; 95% 1.83–4.06) [3]. Moreover, the DBA/2J mouse strain is a very well known model of secondary glaucoma to study neurodegeneration [72] displaying mutations of the genes encoding for the following two proteins: tyrosinase-related protein (TYRP1) and glycosylated transmembrane protein (GPNMB), leading to ocular hypertension for blockade of aqueous outflow by 9 months of age and consequent axonal damage of the optic nerve head [73]. Also, in POAG one of the main targets of treatment is the decrease of IOP to afford neuroprotection. The present HuGe systematic review and meta-analysis aims at clarifying the pharmacogenetic of the therapy of POAG in order to address patients to a better efficacy and safety of treatments. The systematic search retrieved 1022 records, but only 8 met the eligibility criteria, hence pointing at the need for further studies in the field. In particular, it is possible to divide the main pharmacological therapies for which genotypes were subjected to investigation in latanoprost and timolol. The genes most investigated include PTGFR, MRP4 and SNPs of the CYP450, studied mainly to understand susceptibility to be extensive or poor metabolizers, thus experiencing more side effects. The meta-analysis for the efficacy outcome demonstrated statistically significant effect of genetic variants on efficacy outcome (OR 34.80 [9.70–124.88], *P* < 0.00001). On the contrary, the meta-analysis for the safety outcome demonstrated that the effect of SNPs of CYP450 on the risk to develop bradycardia after treatment with timolol was not statistically significant (OR 6.15 [0.37–103.45], *P* = 0.21). A multiethnic GWAS [74] identified the following 24 additional loci causing experimental POAG-like conditions that are not studied in pharmacogenetics. Moreover, among those retrieved, the sole study by Colomb et al. [59] investigated the effect of TIGR/MYOC gene on POAG phenotype on 142 patients, demonstrating that the G allele (MYOC.mt1) is associated with increased impairment of visual field (*P* = 0.02), IOP (+ 4.9 mmHg, *P* = 0.0004) and slower decrease of IOP after therapy with primarily topical beta-blockers that could be associated with miotics. The gene encoding myocilin is fundamental in the pathogenesis of PAOG and it was also used for the production of several lines of transgenic mice for research [75, 76] since it causes IOP elevation. A recent study assessed the influence of 22 genetic variants predisposing to POAG with visual field loss in Japanese patients (n = 426) and control subjects (n = 246), classifying the genotypes into those associated with IOP elevation or with optic nerve vulnerability independent of IOP and assessing indicators of the severity of visual field loss [77]. Therefore, the effect of better response can be due to the baseline difference in IOP caused by the SNP, but the effect of the genotype on all the novel aspects of neuroprotection [78] and on visual loss in the long-term deserves deeper investigation in well-designed studies with homogeneous outcome measures. Furthermore, more clinical trials are needed assessing both the effect of altered metabolism due to genetic variants, but also how safety can be affected by SNPs of genes encoding for proteins involved in pathophysiology of POAG but that can be associated to off target phenomena in other districts. Finally, the involvement of miRNA in the efficacy and safety of the pharmacological treatment of POAG needs to be assessed.

## Data Availability

All data generated or analysed during this study are included in this published article.
